# Determinants of quality of life of cancer patients at a tertiary care medical city in Riyadh, Saudi Arabia

**DOI:** 10.3389/fpsyt.2023.1098176

**Published:** 2023-02-08

**Authors:** Mohammed A. AlJaffar, Sari S. Enani, Ahmad H. Almadani, Fay H. Albuqami, Khalid A. Alsaleh, Fahad D. Alosaimi

**Affiliations:** ^1^Department of Psychiatry, College of Medicine, King Saud University, Riyadh, Saudi Arabia; ^2^Department of Psychiatry, King Saud University Medical City, King Saud University, Riyadh, Saudi Arabia; ^3^Department of Psychiatry, Prince Sultan Military Medical City, Riyadh, Saudi Arabia; ^4^College of Medicine, King Saud University, Riyadh, Saudi Arabia; ^5^Oncology Center, College of Medicine, King Saud University, Riyadh, Saudi Arabia

**Keywords:** cancer, oncology, quality of life, depression, cultural

## Abstract

**Background:**

Incidences of cancer are increasing at an unprecedented rate in Saudi Arabia, making it a major public health concern. Cancer patients are faced with physical, psychological, social, and economic challenges, all of which can impact quality of life (QoL).

**Objectives:**

This study aims to explore the sociodemographic, psychological, clinical, cultural, and personal factors that could affect the overall QoL of cancer patients.

**Methods:**

A total of 276 cancer patients who attended the King Saud University Medical City's oncology outpatient clinics between January 2018 to December 2019 were included. QoL was assessed with the Arabic version of the European Organization for Research and Treatment of Cancer Quality of Life Questionnaire-C30. Psychosocial factors were assessed with several validated scales.

**Results:**

QoL was poorer among patients who were female (*p* = 0.001), have visited a psychiatrist (*p* = 0.028); were taking psychiatric medications (*p* = 0.022); and had experienced anxiety (*p* < 0.001), depression (*p* < 0.001), and distress (*p* < 0.001). The most used method to self-treat was Islamic Ruqya (spiritual healing; 48.6%), and the most often perceived cause for developing cancer was evil eye or magic (28.6%). Good QoL outcomes were associated with biological treatment (*p* = 0.034) and satisfaction with health care (*p* = 0.001). A regression analysis showed that female sex, depression, and dissatisfaction with health care were independently associated with poor QoL.

**Conclusions:**

This study demonstrates that several factors could influence cancer patients' QoL. For instance, female sex, depression, and dissatisfaction with health care were all predictors of poor QoL. Our findings support the need for more programs and interventions to improve the social services for cancer patients, along with the need to explore the social difficulties oncology patients face and address such obstacles through improving social services by expanding the scope of social workers' contribution. Larger multicenter longitudinal studies are warranted to examine the generalizability of the results.

## 1. Introduction

Incidences of cancer have increased greatly over the past decade in Saudi Arabia, making it an emerging burden and a major public health concern (1–5). A prominent factor that influences this increase is the recent socio-economic shift in Saudi communities, such as citizens adopting a far more sedentary lifestyle and consuming more processed foods (2).

Cancer not only affects physical patients' health but also adds a psychosocial burden, which can increase its debilitating effects. For instance, cancer can reduce patients' quality of life (QoL), thereby worsening disease progression and survival rates (6–9). Moreover, cancer patients are more prone to experience depression, anxiety, and fatigue (10). The authors of a meta-analysis concluded that the prevalence of depression in cancer patients ranges between 8 and 24%, as it varies, depending on the type of cancer, treatment phase, and the type of reporting instrument (11). Anxiety proved to be just as prevalent, as 19% of cancer patients had clinical and 22.6% had subclinical levels of anxiety (8). A study conducted in Saudi Arabia in 2022 to assess QoL and the psychological wellbeing of breast cancer survivors reported that 57% of the patients had moderate to severe depression and 44% had moderate to severe anxiety, yet only 6.8% of these patients reported receiving psychosocial support (12). The treatment of cancer can be equally distressing, as it can cause an array of side-effects, including hair loss, pain, and nausea and vomiting, all which impact patients' levels of functioning negatively and cause further deterioration in the mental and physical QoL (6, 10, 13–17).

QoL was defined by Britannica as “the degree to which an individual is healthy, comfortable, and able to participate in or enjoy life events” (18). QoL is a multidimensional concept that measures outcomes in various life domains, which includes physical, role, cognitive, emotional, and social functioning (19). Poor QoL among cancer patients has been reported as high as 82.3% worldwide compared to a recent study done in Saudi Arabia which found that 51% of cancer patients had an overall pool QoL prevalence (15, 20). A systematic review found that the implementation of patient-reported outcome measures can improve communication with cancer patients, enhance treatment response monitoring, and increase patient satisfaction. As a result, it improves fatigue, pain, and loss of appetite among cancer patients (21, 22). Studies conducted in Saudi Arabia have identified various clinical, treatment, and sociodemographic factors that could influence cancer patients' QoL (12, 23–28). Factors such as employment status, educational level, and tumor location were associated with major reductions in all QoL domains (24). More specifically, factors such as age, employment status, income, tumor location, and exercise were associated with social and cognitive functioning, while age, level of education, employment status, and exercise were associated with physical functioning (24–27). Age at diagnosis, level of education, marital status, exercise, psychological program participation, and the Hospital Anxiety and Depression Scale (HADS) scores are predictors of global QoL (12, 24, 26). However, an examination of the impact of other prognostic factors is required to better align the level of care with patients' needs and to enhance the overall wellbeing of cancer patients (28).

Given the significance of the topic and the relative lack of studies on it in Saudi Arabia, the current study aims to identify the sociodemographic, clinical, psychosocial, and cultural factors associated with the QoL of cancer patients.

## 2. Materials and methods

### 2.1. Setting and sampling

This is a cross-sectional study, and the duration designated for data collection was from January 2018 to December 2019. The sample was patients who attended the oncology outpatient clinic at King Saud University Medical City (KSUMC) in Riyadh. The inclusion criteria included adult patients with breast cancer, CRC, and lymphoma, the most common types of cancer encountered in the clinic. Patients diagnosed with other types of cancer or who had cognitive impairment were excluded. Data were collected while patients were attending their regular outpatient appointments. Although the tools of the study were all self-reported, data collectors were around to guide the patients through the process of filling out the study's questionaries. Patients who were observed to be unstable, psychiatric-wise, during the study period were directed to the ER for emergent evaluation and instructed to make urgent appointments with their primary treating psychiatrists, noting that all patients diagnosed with psychiatric issues were already on psychiatric medications and were followed regularly in the psychiatric clinic.

Concerning the sample size calculation, the Raosoft^®^ software was used to calculate the required sample size based on the estimated parameters of the study population. For a total target population of 1,000 patients, with a 95% confidence level and a 5% margin of error, the sample size was estimated to be 278. Comparable studies in this regard used sample sizes ranging between 182 and 345, while locally sample sizes ranged between 159 and 393 (20, 29–34).

All patients signed an informed consent form and then completed the questionnaire anonymously. Patients were not offered rewards or incentives for their participation. Ethical approval was obtained from the Institutional Review Board of the College of Medicine at King Saud University (project #E-17-2769).

### 2.2. Questionnaire

The questionnaire is comprised of four sections, namely (i) patient demographics, including sociodemographic background, medical and psychiatric history, personal beliefs regarding cancer etiology, and religious or alternative remedies; (ii) assessment of QoL; (iii) screening for depression, anxiety, and distress; and (iv) satisfaction related to health care and social support.

QoL was assessed using the previously validated Arabic language version of the European Organization for Research and Treatment of Cancer Quality of Life Questionnaire-C30 (EORTC QLQ-C30) (35) which showed good internal reliability with six out of nine scales displaying Cronbach's alpha coefficients of >0.70 (36). The Arabic (EORTC QLQ) is composed of five functional subscales (physical, role, cognitive, emotional, and social), three symptom subscales (fatigue, pain, and nausea and vomiting), and a Global Health Status and Quality of Life Scale (GHS/QoL) (36). Most items are scored from 1 to 4, except for items that contribute to GHS/QoL, which are assessed on a 7-point scale. All scale measures are transposed to a 100-point scale, where a higher score represents a higher response level. Hence, a high score on a functional subscale indicates a healthier level of functioning, a high score on the global health status indicates a high QoL, and a high score on a symptom subscale indicates a high level of symptomatology/problems (36). The Cronbach's alpha of the Arabic (EORTC QLQ) questionnaire was found to be more than 0.70 for six out of its nine subscales (36).

We also screened patients for depression and anxiety using the Patient Health Questionnaire 9-item (PHQ-9) and the Generalized Anxiety Disorder 7-item (GAD-7) questionnaires, respectively (37). The Arabic version of the PHQ was found to be valid and reliable for the screening of many psychiatric disorders, including depression (PHQ-9) and anxiety (GAD-7), as it exhibits good internal consistency, with a Cronbach's alpha score of 0.857 (37). A cutoff score of ≥10 was used to define depression (in PHQ-9) and anxiety (in GAD-7), as calculated using the 75% quartile and consistent with previous studies (38, 39). Satisfaction toward health care and social support were scored on a 10 mm visual analog scale (VAS) (40). The VAS is a horizontal line numbered from 1 to 10 and is used for self-reporting of satisfaction (40). A VAS score > 8 was considered “satisfied” using the 75% quartile, consistent with a study by Kawai et al. (40).

Furthermore, distress was measured using the Distress Thermometer (DT), which was originated by the National Comprehensive Cancer Network (NCCN) to evaluate distress in oncology patients. This instrument is used to measure distress on a 0–10 scale, with higher scores indicating greater distress (41). The validated Arabic version of the DT was used which had a (0.63) (0.70) specificity and sensitivity respectively, with a cut-off score of ≥4, which indicates distress (41).

### 2.3. Statistical analysis

The data were analyzed using Statistical Packages for Social Sciences (SPSS) version 26 (Armonk, NY: IBM Corp). Continuous variables are presented as the mean and standard deviation, while categorical variables are presented as numbers and percentages. QoL was categorized as poor QoL (≤ 60%) and good QoL (>60%), in accordance with a study by Derogar et al. (42). QoL was compared across sociodemographic clinical and psychosocial characteristics, using the chi-square test and Mann–Whitney *U*-test, as appropriate. Normality tests were performed using the Shapiro–Wilk test, where non-parametric tests were performed on continuous data deemed to be non-normal distribution. Significant results were then placed in a multivariate regression model, and the odds ratio and 95% CI were also reported. A *p*-value of 0.05 at 95% CI was used to determine statistical significance.

## 3. Results

Two hundred and seventy-six cancer patients participated in this study. Most patients were older than 50 years (56.9%), nearly two-thirds were females (63.8%), and most patients were Saudi (84.1%). The majority of patients were married (82.2%), while nearly three-quarters (72.1%) were unemployed. Most patients (59.1%) had a high school or lower level of education, and 62.3% had a monthly income of 10,000 SAR or less. Furthermore, 70.3% were living in owned residences, mainly in the Central Region (79.7%). The only sociodemographic factor statistically linked to QoL was sex, as QoL was significantly worse among females (*X*^2^ = 11.149; *p* = 0.001; see Table 1).

**Table 1:** Sociodemographic characteristics of the patients and their quality of life.

**Study data**	**Overall**	**Quality of life**		***P*-value^§^**
	***N*** **(%) (*****n*** = **276)**	**Poor**	**Good**	
		***N*** **(%) (*****n*** = **74)**	***N*** **(%) (*****n*** = **202)**	
**Age group**				
• ≤ 50 years	119 (43.1%)	38 (51.4%)	81 (40.1%)	0.095
• >50 years	157 (56.9%)	36 (48.6%)	121 (59.9%)	
**Sex**				
• Male	100 (36.2%)	15 (20.3%)	85 (42.1%)	**0.001** ^**^
• Female	176 (63.8%)	59 (79.7%)	117 (57.9%)	
**Nationality**				
• Saudi	232 (84.1%)	60 (81.1%)	172 (85.1%)	0.414
• Non-Saudi	44 (15.9%)	14 (18.9%)	30 (14.9%)	
**Marital status**				
• Unmarried	49 (17.8%)	17 (23.0%)	32 (15.8%)	0.170
• Married	227 (82.2%)	57 (77.0%)	170 (84.2%)	
**Occupation**				
• Employed	77 (27.9%)	19 (25.7%)	58 (28.7%)	0.618
• Unemployed	199 (72.1%)	55 (74.3%)	144 (71.3%)	
**Educational level**				
• High school or below	163 (59.1%)	50 (67.6%)	113 (55.9%)	0.082
• Bachelor or higher	113 (40.9%)	24 (32.4%)	89 (44.1%)	
**Monthly income (SAR)**				
• ≤ 10,000	172 (62.3%)	50 (67.6%)	122 (60.4%)	0.276
• >10,000	104 (37.7%)	24 (32.4%)	80 (39.6%)	
**Type of residence**				
• Owned	194 (70.3%)	51 (68.9%)	143 (70.8%)	0.763
• Rented	82 (29.7%)	23 (31.1%)	59 (29.2%)	
**Region**				
• Central	220 (79.7%)	58 (78.4%)	162 (80.2%)	0.739
• Non-central	56 (20.3%)	16 (21.6%)	40 (19.8%)	

§ *P*-value was calculated using the chi-square test.

** Significant at the *p* < 0.05 level.

Bold means significant at the p < 0.05 level.

The clinical characteristics of the patients with cancer are shown in Table 2. It was revealed that the most commonly diagnosed cancer was breast cancer (43.8%), and stage 2 was the most common stage (16.3%). Furthermore, the most common intervention was chemotherapy (85.2%). The proportion of patients who visited psychiatrists was 8.7%, while 6.5% have been diagnosed with psychiatric disorders. All patients diagnosed with psychiatric problems were on psychiatric medications. The most commonly diagnosed psychiatric disorder was depression (73.3%), followed by adjustment (13.3%), stress (6.7%), and anxiety (6.7%). The method mostly used to self-treat was Islamic Ruqya (spiritual healing; 48.6%), and the cause of the cancer most often perceived was the evil eye or magic (28.6%) (43). In addition, the overall mean score for satisfaction toward health care was 8.78 (SD 1.56), while the overall mean score for satisfaction toward social support was 8.82 (SD 1.71). In the comparison to QoL, we found that the prevalence of poor QoL was significantly higher among patients who visited a psychiatrist (*X*^2^ = 4.847; *p* = 0.028) or were taking psychiatric medication (*X*^2^ = 5.276; *p* = 0.022), while the prevalence of good QoL was significantly higher among patients who had received biological treatment (*X*^2^ = 4.474; *p* = 0.034) and who had reported satisfaction toward health care (*U* = 5,589.5; *p* = 0.001).

**Table 2:** Clinical characteristics of patients with cancer in relation to quality of life.

**Variables**	**Overall**	**Quality of life**		***P*-value^§^**
	***N* (%) (*n* = 276)**	**Poor**	**Good**	
		***N* (%) (*n* = 74)**	***N* (%) (*n* = 202)**	
**Type of cancer**				
• Breast	121 (43.8%)	41 (55.4%)	80 (39.6%)	0.063
• Colon	111 (40.2%)	24 (32.4%)	87 (43.1%)	
• Lymphoma	44 (15.9%)	09 (12.2%)	35 (17.3%)	
**Stage of cancer**				
• Undetermined	142 (51.4%)	36 (48.6%)	106 (52.5%)	0.184
• Stage 1	24 (08.7%)	05 (06.8%)	19 (09.4%)	
• Stage 2	45 (16.3%)	10 (13.5%)	35 (17.3%)	
• Stage 3	37 (13.4%)	16 (21.6%)	21 (10.4%)	
• Stage 4	28 (10.1%)	07 (09.5%)	21 (10.4%)	
**Type of intervention** ^*^				
• Chemotherapy	236 (85.2%)	59 (79.7%)	177 (87.6%)	0.099
• Radiotherapy	84 (30.4%)	24 (32.4%)	60 (29.7%)	0.662
• Surgery	146 (52.9%)	33 (44.6%)	113 (55.9%)	0.094
• Hormonal therapy	32 (11.6%)	08 (10.8%)	24 (11.9%)	0.806
• Biological	34 (12.3%)	04 (05.4%)	30 (14.9%)	**0.034** ^**^
**Psychiatrist visit**				
• No	252 (91.3%)	63 (85.1%)	189 (93.6%)	**0.028** ^**^
• Yes	24 (08.7%)	11 (14.9%)	13 (06.4%)	
**Diagnosed with psychiatric disorder**				
• No	258 (93.5%)	66 (89.2%)	192 (95.0%)	0.081
• Yes	18 (06.5%)	08 (10.8%)	10 (05.0%)	
**Taking psychiatric medication**				
• No	258 (93.5%)	65 (87.8%)	193 (95.5%)	**0.022** ^**^
• Yes	18 (06.5%)	09 (12.2%)	09 (04.5%)	
**Used of other method to treat the disease**				
• No	120 (43.5%)	30 (40.5%)	90 (44.6%)	0.911
• Islamic Ruqya	134 (48.6%)	37 (50.0%)	97 (48.0%)	
• Alternative/Traditional	06 (02.2%)	02 (02.7%)	04 (02.0%)	
• Certain diet	16 (05.8%)	05 (06.8%)	11 (05.4%)	
**Perceived reason for having a cancer**				
• Don't know	97 (35.1%)	19 (25.7%)	78 (38.6%)	0.168
• Evil eye or Magic	79 (28.6%)	20 (27.0%)	59 (29.2%)	
• Genetics	46 (16.7%)	15 (20.3%)	31 (15.3%)	
• Health condition	42 (15.2%)	16 (21.6%)	26 (12.9%)	
• Other	12 (04.3%)	04 (05.4%)	08 (04.0%)	
**Satisfaction**	**Mean** ±**SD**	**Mean** ±**SD**	**Mean** ±**SD**	***P*** **-value** ^‡^
• Health care	8.78 ± 1.56	8.15 ± 1.98	9.01 ± 1.30	**0.001** ^**^
• Social support	8.82 ± 1.71	8.38 ± 2.41	8.99 ± 1.34	0.368

* Variable with multiple response answers.

§ *P*-value was calculated using the chi-square test.

‡ *P*-value was calculated using the Mann–Whitney U-test.

** Significant at the *p* < 0.05 level.

Bold means significant at the *p* < 0.05 level.

The assessment of the psychological factors in relation to QoL is shown in Table 3. It was found that the prevalence of patients with anxiety, depression, and distress was 21.7, 19.6, and 38.4%, respectively. Poor QoL was more common among patients with anxiety (*X*^2^ = 27.482; *p* < 0.001), depression (*X*^2^ = 44.713; *p* < 0.001), and distress (*X*^2^ = 33.057; *p* < 0.001).

**Table 3:** Assessment of psychological factors in relation to quality of life among cancer patients.

**Psychological factors**	**Overall**	**Quality of life**		***P*-value^§^**
	***N (%)* (*n* = 276)**	**Poor**	**Good**	
		***N (%)* (*n* = 74)**	***N (%)* (*n* = 202)**	
**Anxiety**				
• Anxious (score < 10)	60 (21.7%)	32 (43.2%)	28 (13.9%)	<0.001^**^
• Not anxious (score ≥ 10)	216 (78.3%)	42 (56.8%)	174 (86.1%)	
**Depression**				
• Depressed (score < 10)	54 (19.6%)	34 (45.9%)	20 (09.9%)	<0.001^**^
• Not depressed (score ≥ 10)	222 (80.4%)	40 (54.1%)	182 (90.1%)	
**Distressed**				
• No	170 (61.6%)	25 (33.8%)	145 (71.8%)	<0.001^**^
• Yes	106 (38.4%)	49 (66.2%)	57 (28.2%)	
***Quality of life domains*** **(EORTC QLQ-C30)**	**Mean** ±**SD**	**Mean** ±**SD**	**Mean** ±**SD**	***P*** **-value** ^‡^
	**Scale (100)**	**Scale (100)**	**Scale (100)**	
**Functional scales**	77.1 ± 20.8	58.4 ± 21.2	83.9 ± 15.9	<0.001^**^
• Physical functioning	80.6 ± 17.8	66.1 ± 18.3	85.9 ± 14.4	<0.001^**^
• Role functioning	83.1 ± 20.4	68.6 ± 23.5	88.4 ± 16.3	<0.001^**^
• Cognitive functioning	82.9 ± 26.8	68.5 ± 28.1	88.1 ± 21.3	<0.001^**^
• Social functioning	81.2 ± 26.8	62.8 ± 32.7	87.9 ± 20.6	<0.001^**^
**Symptoms scales**	29.9 ± 24.9	50.8 ± 24.3	22.4 ± 20.4	<0.001^**^
• Fatigue	36.9 ± 28.8	58.3 ± 27.5	29.1 ± 25.2	<0.001^**^
• Pain	27.5 ± 30.7	52.3 ± 32.2	18.5 ± 24.6	<0.001^**^
• Financial difficulties	18.5 ± 31.2	34.2 ± 34.4	50.8 ± 24.3	<0.001^**^

EORTC QLQ-C30, European Organization for Research and Treatment of Cancer Quality of Life Questionnaire.

§ *P*-value was calculated using the chi-square test.

‡ *P*-value was calculated using the Mann–Whitney U-test.

** Significant at the *p* < 0.05 level.

The mean score of the QoL functional scales was 77.1 (SD 20.8). Among its dimensions, the mean scores for physical, role, cognitive, and social functioning were 77.1, 80.6, 83.1, 82.9, and 81.2, respectively. Likewise, the mean score for symptoms scales was 29.9 (SD 29.9). The mean scores for fatigue, pain, and financial difficulties were 36.9, 27.5, and 18.5, respectively. The mean scores of the functional scales (*U* = 2,472; *p* < 0.001), with physical functioning (*U* = 2863; *p* < 0.001), role functioning (*U* = 3,647; *p* < 0.001), cognitive functioning (*U* = 4,377; *p* < 0.001), and social functioning (*U* = 3,939; *p* < 0.001) were significantly higher among patients with good QoL, while the mean scores of the symptom scales (*U* = 2,876; *p* < 0.001), fatigue (*U* = 3,315.5; *p* < 0.001), and pain (*U* = 3,252; *p* < 0.001), were significantly higher among patients with poor QoL. Interestingly, the mean score of financial difficulties was significantly higher among patients with good QoL (*U* = 4,646; *p* < 0.001).

A multivariate regression analysis was subsequently performed to determine the independent predictors associated with poor QoL. Based on the results, on the one hand, it was found that female sex, depression, and distress were independently associated with poor QoL. Moreover, satisfaction with healthcare was associated with good QoL. Female patients had a 2-fold higher risk of poorer QoL than male patients (AOR = 2.912; 95% CI = 1.394–6.081; *p* = 0.004). We also observed that patients with depression (AOR = 3.201; 95% CI = 1.410–7.265; *p* = 0.005) and distressed patients (AOR = 3.290; 95% CI = 1.682–6.438; *p* = 0.001) were three times more likely to have poor QoL, whereas satisfaction with health care was associated with good QoL (AOR = 1.284; 95% CI = 1.060–1.556; *p* = 0.011). On the other hand, biological intervention, treatment with psychiatric medication, and anxiety did not show a significant effect after adjustment of the regression model (*p* > 0.05; see Table 4).

**Table 4:** Multivariate regression analysis to determine the factors associated with poor quality of life among cancer patients.

**Factor**	**AOR**	**95% CI**	***P*-value**
**Sex**			
• Female	Ref 2.912	1.394–6.081	**0.004** ^**^
**Biological intervention**			
• Yes	Ref 0.341	0.101–1.157	0.084
**Psychiatrist visit**			
• Yes	Ref 0.282	0.034–2.326	0.240
**Taking psychiatric medication**			
• Yes	Ref 2.686	0.272–26.536	0.398
**Anxiety**			
• Anxious	Ref 1.516	0.636–3.610	0.348
**Depression**			
• Depressed	Ref 3.201	1.410–7.265	**0.005** ^**^
**Distressed**			
• Yes	Ref 3.290	1.682–6.438	**0.001** ^**^
**Satisfaction with health care**			
• No	Ref 1.284	1.060–1.556	**0.011** ^**^

AOR, adjusted odds ratio; CI, confidence interval.

** Significant at the p < 0.05 level.

Bold means significant at the *p* < 0.05 level.

## 4. Discussion

The current study focuses on factors associated with QoL among cancer patients. Previous studies have concluded that age, education, and economic status were associated with QoL (16, 44, 45). The results of this study revealed that QoL was not influenced by any demographic characteristics or socioeconomic status, except for female sex. However, depression and distress were found to be associated with poor QoL, while satisfaction with healthcare was associated with good QoL.

This study found that all domains of functioning were impaired among patients with poor QoL, which aligns with findings by Hinz et al. (46). Among such patients in this study, social functioning was found to be the most impaired subdomain. Previous studies have emphasized the importance of social support for cancer patients, which has a great impact on QoL (47, 48).

In this study, patients with poor QoL suffered from increased fatigue. Fatigue is the most commonly experienced side effect of cancer and cancer treatment (13, 49, 50). In addition, it is more common in older patients and among those with a lower socioeconomic status (50). It has been demonstrated that increased levels of fatigue are not only associated with ongoing chemotherapy sessions but can also last for several years after the completion of chemotherapy (13, 49, 51). However, many patients experiencing fatigue do not receive any support from healthcare providers to reduce their fatigue (13).

The findings of this study show that poor QoL is associated with a decrease in physical function and an increase in fatigue. Cancer patients should be encouraged to exercise regularly, as exercise can decrease fatigue and improve QoL (52). In this study, the pain scores of patients with poor QoL were found to be considerably higher than the mean scores of the other patients. The thinking is that pain has a negative impact on patients' psychological health, sleep, and daily activities, which, subsequently, decreases their QoL (53). In addition, breast cancer patients with metastases had worse QoL and greater pain scores (54).

The present study found that patients with depression were three times more likely to have impaired QoL, a finding that is supported by the findings of other studies (47, 55). For instance, it was found in a meta-analysis that the prevalence of depression in patients with cancer ranged from 8 to 24% (11). The prevalence of depression in the present study was 19.6%, but only 8.7% of the patients had visited a psychiatric clinic. Therefore, screening for depression and other psychological symptoms in cancer patients and referrals to specialists are strongly advised. Moreover, female patients had a 2-fold higher risk of a poorer QoL. Studies have found that female patients had worse mental QoL than males, and depression is more common among women (17, 28, 45, 56). Hence, women who have poorer QoL may also have mental-related symptoms.

Interestingly, almost one third of the present study's patients attributed their cancer to supernatural causes, such as the evil eye or magic, and almost half used Islamic Ruqya (spiritual healing); however, these factors were not associated with the level of their QoL. These findings are consistent with a recent Saudi study that reported the trends of the perceptions related to the causes of cancer between 2008 and 2018, in which the authors found a significant increase, from 1.3 to 33.1%, in the belief of the “evil eye” as a cause of cancer (57). Another study on the use of complementary and alternative medicines among patients with cancer in Saudi Arabia found that patients were using Zamzam water (59.8%), honey (54.3%), black seed (35.1%), and water that had the Quran recited over it (29.8%) (58). These findings emphasized the strong influence of religion on peoples' lives, especially when confronted with life-threatening illnesses. Therefore, a systematic approach toward educating patients and the public as well as the licensing of spiritual cancer care workers to formalize their practice through education and training might be required to improve patient care and patient outcomes (57). Overall, the assessment of spiritual/religious needs can be considered as the first step in designing needs-tailored interventions (59).

This study has some limitations. First, the study's sample was recruited from one center. Hence, further multicenter studies are warranted to examine the generalizability of the results. Second, we only included patients with breast cancer, CRC, and lymphoma. Hence, other studies exploring other types of cancer could further enrich our understanding of the QoL of cancer patients. Third, this study is a cross-sectional study. Thus, future research could extend this work by conducting a longitudinal study. Finally, it was difficult to determine the prevalence of the factors associated with poor quality of life given the heterogenicity of psychosocial and clinical variables associated with poor quality of life therefore. Further studies should consider enough sample size to measure the psychosocial and clinical variables associated with poor quality of life among cancer patients.

Despite these limitations, this study provides valuable insights into cancer patients' QoL and the many factors impacting it. Addressing such factors in healthcare can improve the wellness and overall health of patients, especially cancer patients.

## 5. Conclusions

Our study aimed to identify the sociodemographic, clinical, psychosocial, and cultural factors associated with the QoL of cancer patients. Concerning the sociodemographic aspect, the female gender was an independent factor which contributed to the poor QoL. Among contributing clinical factors, our results indicated that pain, fatigue, and dissatisfaction with health care all significantly affect the QoL among cancer patients. The only psychosocial factor contributing to poor QoL was depression. Our findings support the need for more programs and interventions to improve the social services for cancer patients, along with the need to explore the social difficulties oncology patients face and address such obstacles through improving social services by expanding the scope of social workers' contribution.

## Data availability statement

The original contributions presented in the study are included in the article/supplementary material, further inquiries can be directed to the corresponding author.

## Ethics statement

The studies involving human participants were reviewed and approved by the Institutional Review Board of the College of Medicine at King Saud University (project#E-17-2769). The patients/participants provided their written informed consent to participate in this study.

## Author contributions

All authors made substantial contributions to conception and design, acquisition of data, analysis and interpretation of data, took part in drafting the article or revising it critically for important intellectual content, agreed to submit it to the current journal, gave final approval to the version to be published, and agree to be accountable for all aspects of the work.
